# Potential associations of food allergy and altered neurodevelopment in children

**DOI:** 10.1192/j.eurpsy.2023.732

**Published:** 2023-07-19

**Authors:** B. Abdelmoula, N. Kharrat, R. Abdelhedi, N. Bouayed Abdelmoula

**Affiliations:** 1 Genomics of Signalopathies at the service of Medicine, Medical University of Sfax; 2 Research group on molecular and screening process; 3 Research group on molecular and screening process and, Centre of Biotechnology of Sfax, Sfax, Tunisia

## Abstract

**Introduction:**

Allergic immune reactions and adverse reactions to foods, have been described as having growth concerns in children with food allergy. Moreover, immune dysregulation and inflammation have been documented as typical hallmarks in both allergic and neurodevelopmental conditions.

**Objectives:**

In this review, we address the association of food allergy and altered neurodevelopment in children.

**Methods:**

We comprehensively review the scientific literature using Pubmed database and other search platforms to state the potential associations of food allergy and altered neurodevelopment in children.

**Results:**

Food allergy is a pathological, potentially deadly, immune reaction activated by normally inoffensive food protein antigens. It is an important public health problem that affects children (children under the age of 5 years: 5 %) and adults, and it has been increasing in prevalence in the last 2 to 3 decades. The enhancement of the knowledge of the pathophysiological mechanisms lead to many suggestions such as the important role of the intestinal microbiota, the role of the immunological adaptation of the mucosal immune system to food antigens and the nutritional impact and growth concerns of children with food allergy. In recent studies and reviews, a significant and a positive association of common allergic conditions, in particular food allergy, with autism spectrum disorder and with attention deficit hyperactivity disorders have been reported. At the mechanistic level, it was recently shown through animal models, the potential role of intracranial Mast cells in neuroinflammation and neuropathology associated with food allergy as well as the potential role of the dysfunction of the gut-brain axis in promoting white matter development during early life when the brain is vulnerable to environment (such as food restrictions) that can result in an a wide spectrum of neurodevelopmental disorders later in life. Food allergy was also associated in literature with enhanced mTOR signaling in the brain and gut, which may impact brain and behavioral development.

**Conclusions:**

Neurodevelopmental disorders which occur in childhood in the context of food allergy is a challenging public health problem that need more human research studies to understand underlying mechanisms and promote therapeutic innovations.

**Disclosure of Interest:**

None Declared

